# Evidences and perspectives of transjugular intrahepatic portosystemic shunt: A bibliometric analysis from 1990 to 2024

**DOI:** 10.1097/MD.0000000000046229

**Published:** 2025-11-28

**Authors:** Kejia Li, Wenqing Zhang, Fan Yang, Lei Zhang, Songwei Li

**Affiliations:** a Department of Pharmacy, The Second Affiliated Hospital of Kunming Medical University, Kunming, Yunnan Province, China; b Department of Interventional Radiology, The Second Affiliated Hospital of Kunming Medical University, Kunming, Yunnan Province, China.

**Keywords:** bibliometric analysis, CiteSpace, frontiers, hotspots, transjugular intrahepatic portosystemic shunt, VOSviewer

## Abstract

**Background:**

Transjugular intrahepatic portosystemic shunt (TIPS) is the mainstay of cirrhotic portal hypertension therapy. The extension of TIPS indications is relatively prevalent in clinical practice these years, but its prognosis is still controversial. However, no bibliometric study in this area has been performed to date.

This study aimed to provide insights and perspectives on the development, hotspots and frontiers of TIPS.

**Methods:**

A total of 3152 related articles from 1990 to 2024 were retrieved from the Web of Science Core Collection database, and assessed using VOSviewer (version 1.6.19), CiteSpace (version 6.3.R1) and Bibliometrix R-package (version 4.3.0).

**Results:**

The United States was the leading country contributing to this field, and the University of Barcelona stood out as the most prominent institution. *Journal of Vascular and Interventional Radiology* ranked first in publication, while *Hepatology* received the highest citation frequency. Trebicka Jonel’s publication numbers and citations were both on the top ten author list, and demonstrated great total link strength. During this period, “hepatic encephalopathy” was regarded as a persistent hotspot, while “sarcopenia” emerged as the new frontier in the research area.

**Conclusion:**

We hypothesize that indication extension, optimal patient selection and the time of intervention will become the main concerns in the near future.

## 1. Introduction

Portal hypertension is the prominent complication of cirrhosis, contributing to severe complications and even mortality.^[1]^ Transjugular intrahepatic portosystemic shunt (TIPS) reduces portal pressure via creating an artificial channel between the hepatic and portal vein, and subsequently, alleviates and prevents variceal bleeding and refractory ascites. Thus, TIPS is recommended as the mainstay of cirrhotic portal hypertension therapy.^[2]^ Since its clinical introduction in 1988, TIPS has undergone significant advancements, particularly the use of specialized purpose-designed polytetrafluoroethylene-covered stents over the past 2 decades.^[3]^ In recent years, the extension of TIPS indications is relatively prevalent in clinical practice, but the prognosis of those patients with extended indications is still controversial. Therefore, it is imperative to synthesize the current evidence and future trends in this field.

Bibliometrics is an essential tool to synthesize and analyze the entire corpus of literature quantitatively and qualitatively, which integrates statistical techniques with information visualization technology.^[4]^ Since first published in 1975,^[5]^ interest in bibliometrics has been booming with the iteration of analytic approaches. Given its comprehensive statistical power, bibliometrics is becoming more and more widely available in the biomedical fields nowadays.^[6]^ To our knowledge, no bibliometric study has focused on TIPS to date.

Our study provided insights and perspectives on the development, hotspots and frontiers of TIPS via bibliometric analysis based on Web of Science Core Collection (WoSCC) database, with a view to shedding light on the indication and timing of TIPS, and identify potential future trends in this field.

## 2. Methods

### 2.1. Document source and search strategy

Scientific literature published from January 1, 1990 to December 31, 2024 was retrieved from WoSCC database on the same day to avoid database update bias, using the following formula: TS= (“transjugular intrahepatic portosystemic shunt”) OR (“transjugular intrahepatic portosystemic stent-shunt”). The types of literature were restricted to “articles” or “reviews,” and the language of publication was restricted to English. Duplicate literature was excluded. The data were exported as “full-text records and references” and saved as “plain text” files. Data extraction was conducted independently by 2 researchers, and proofread by the third researcher.

### 2.2. Statistical analysis and visualization

**Figure 1.**
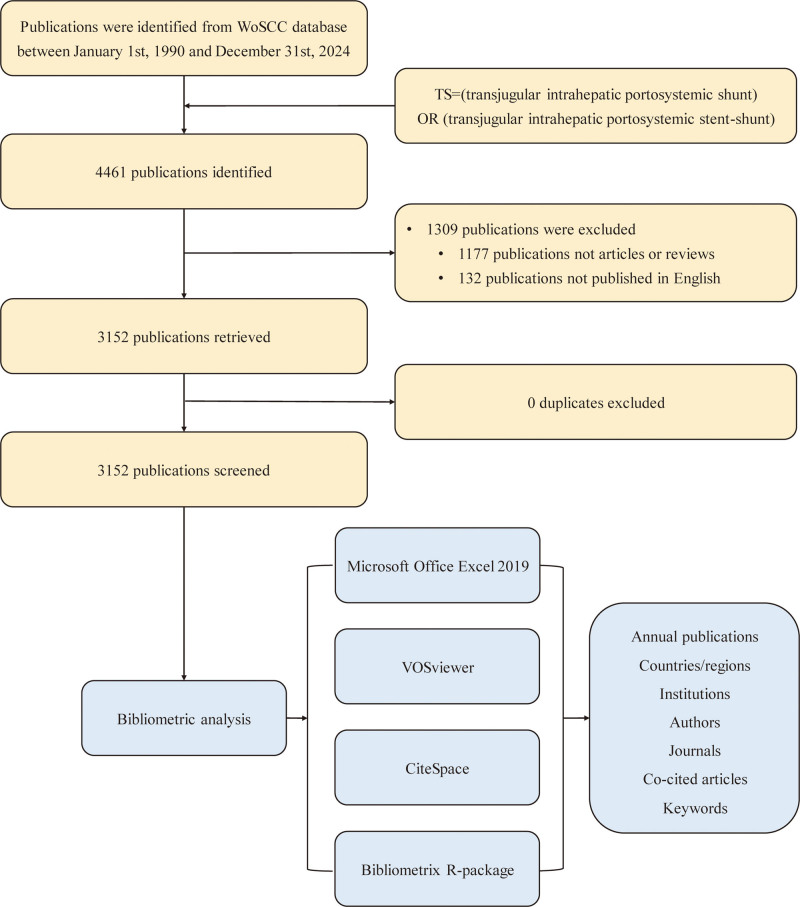
Flow chart of the study.

EndNote was used for removing the duplicates, and Microsoft Office Excel 2019 was used for data entry and general charts depicting. VOSviewer (version 1.6.19),^[7]^ CiteSpace (version 6.3.R1)^[8]^ and Bibliometrix R-package (version 4.3.0)^[9]^ were used for bibliometric analysis and data visualization, including geographic distribution of national collaborations, institutional collaboration networks, trends in journal publications, citation patterns, co-occurrence of keywords, keyword clustering and changes in hot research topics. Detailed processes of literature search and bibliometric analysis is displayed in Figure 1.

## 3. Results

### 3.1. The annual publication trend

**Figure 2.**
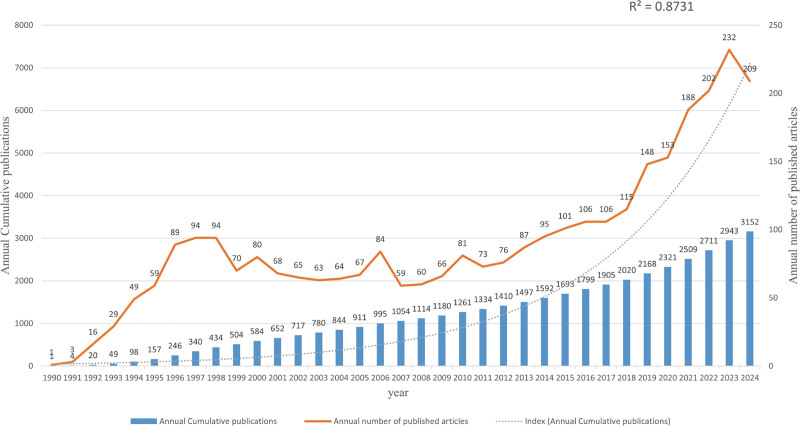
The annual publication trend about TIPS. TIPS = transjugular intrahepatic portosystemic shunt.

A total of 3152 related articles were retrieved from WoSCC database. Trend analysis indicated a linear increase in cumulative publication, and a 3-phase growth in annual publication during 1990 to 2024. Since the first TIPS was placed, the amount of literature surged (1990–1997), and reached a plateau between 1998 and 2012, and then underwent exponential growth again from 2013. In the initial 2 stage, 1410 articles were published, accounting for just 44.7% of the total. In the third stage, annual publication maintained steady growth at an average rate of ~9.2% per year, and attained its peak in 2023 (232 articles) (Fig. 2).

### 3.2. Contributions of countries/regions to global publications

Among the 78 countries/regions publishing papers on TIPS, the United States ranked first in publication number at 1059 (33.6%), followed by China at 528 (16.8%) and Germany at 357 (11.3%). In terms of citations, the United States was still at the top, with 37,545 citations, and the number of citations from Italy, Germany and Spain were also over ten thousand. Notably, literature from Netherlands had the highest average article citations rate at 107.14 citations per paper (37 publications with 3964 citations) (Table 1).

**Table 1:** The top 10 countries/regions contributing the most to the field.

Rank	Country	Counts	Country	Total citations	Country	Average article citations	Country	Total link strength
1	America	1059	America	37,545	Netherlands	107.14	Spain	265
2	China	528	Italy	14,997	Italy	68.79	Germany	259
3	Germany	357	Germany	14,579	Spain	66.45	America	224
4	United Kingdom	220	Spain	12,691	Canada	63.00	Italy	215
5	Italy	218	United Kingdom	8617	Denmark	60.51	France	172
6	Spain	191	France	8463	France	57.57	Switzerland	134
7	France	147	China	6461	Switzerland	42.47	Denmark	132
8	Canada	93	Canada	5859	Germany	40.84	United Kingdom	114
9	India	86	Netherlands	3964	Belgium	40.83	Belgium	114
10	Belgium	78	Denmark	3570	United Kingdom	39.17	Canada	106

**Figure 3.**
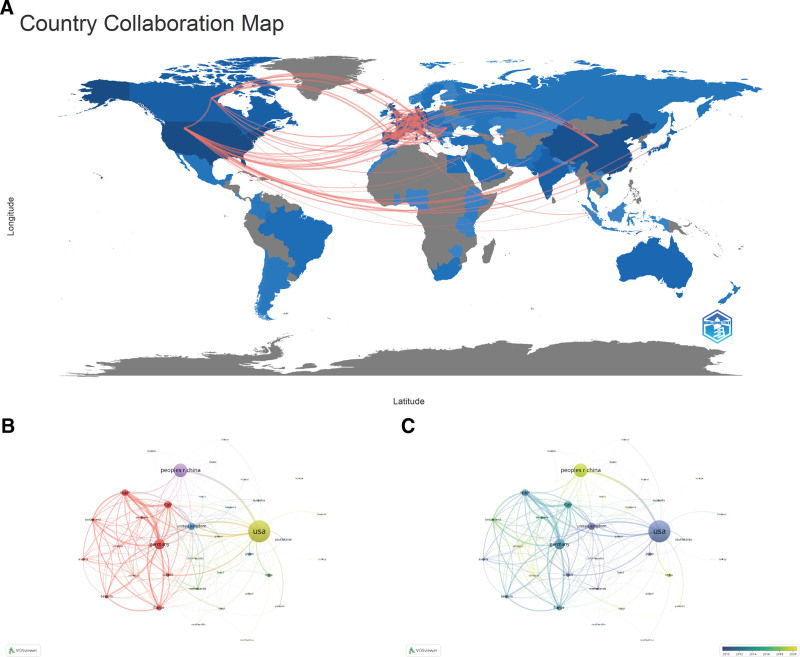
Geographical distribution and cooperation network of countries/regions in the field. (A) The country collaboration map. The width of the lines reflected the frequency of collaboration between the 2 countries/regions. (B) The country collaboration network. The size of the nodes and the width of the connecting lines represented the publication number and cooperation strength of the countries/regions, respectively. (C) The temporal overlay network map of countries/regions. The brighter the nodes, the more recent the average year of publications.

The country collaboration map showed that the United States undoubtedly cooperated with other countries most closely and extensively, and relatively, the scholarly communication within Europe seemed more deeply and frequently (Fig. 3A, B). However, as illustrated in temporal overlay network map, China was gradually taking the lead in international collaboration these years, and some countries in South Asia and Eastern Europe became more active as well (Fig. 3C).

### 3.3. Institutions for research on TIPS

**Figure 4.**
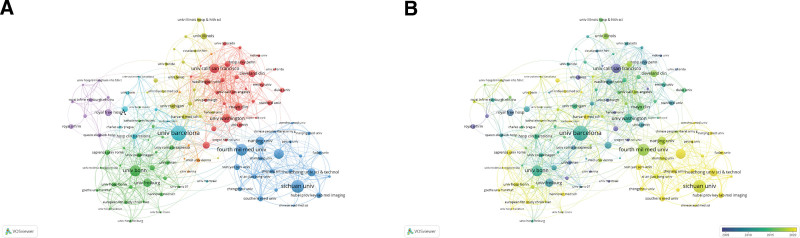
Distribution and cooperation network of institutions in research on TIPS. (A) The institution collaboration network. The size of the nodes and the width of the connecting lines represented the publication number and cooperation strength of the institutions, respectively. (B) The temporal overlay network map of institutions. The brighter the nodes, the more recent the average year of publications. TIPS = transjugular intrahepatic portosystemic shunt.

Among the 2472 institutions contributing to this field, University of Barcelona in Spain was undoubtedly the most influential one, with 72 papers, 5808 citations and the strongest link with other institutions. Within the top 10 list of publication number, there were 5 institutions from China, 3 from Europe, and 2 from the United States, while more than half of the top 10-cited institutions were from Europe (Table 2). As illustrated in Figure 4, institutions in Europe and North America maintained ongoing collaboration over the entire research period, whereas Chinese institutions engaged in within-region collaboration actively in recent years.

**Table 2:** The top ten institutions for research on TIPS.

Rank	Institution	Counts	Institution	Total citations	Institution	Total link strength
1	University of Barcelona (Spain)	72	Mayo Clinic (USA)	6501	University of Barcelona (Spain)	114
2	Sichuan University (China)	62	University of Barcelona (Spain)	5808	University of Bonn (Germany)	108
3	The Fourth Military Medical University (China)	57	Hospital Clinic Barcelona (Spain)	4094	The Fourth Military Medical University (China)	106
4	University of Bonn (Germany)	52	University of Bonn (Germany)	3551	Nanjing University (China)	87
5	Capital Medical University (China)	47	University Freiburg (Germany)	3517	Hospital Clinic Barcelona (Spain)	85
6	University of California, San Francisco (USA)	45	Beth Israel Deaconess Medical Center (Israel)	2811	Northwestern University (USA)	81
7	University of Washington (USA)	43	University of California, San Francisco (USA)	2471	Southern Medical University (China)	72
8	Nanjing University (China)	40	The Fourth Military Medical University (China)	2457	Huazhong University of Science and Technology (China)	70
9	University of Freiburg (Germany)	40	University of Toronto (Canada)	2431	Zhejiang University (China)	67
10	Huazhong University of Science and Technology (China)	39	University of Copenhagen (Denmark)	2379	Shandong University (China)	64

TIPS = transjugular intrahepatic portosystemic shunt.

### 3.4. Author’s impact in this field

**Figure 5.**
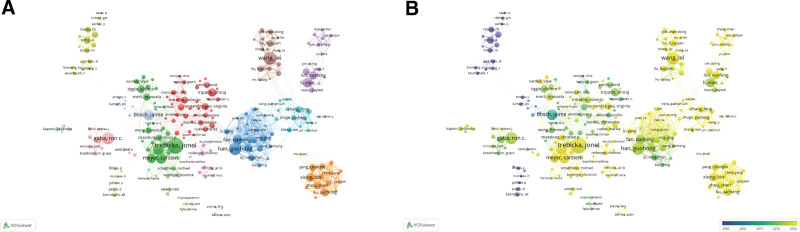
Cooperation network among authors in research on TIPS. (A) The author collaboration network. The size of the nodes and the width of the connecting lines represented the publication number and cooperation strength of the authors, respectively. (B) The temporal overlay network map of authors. The brighter the nodes, the more recent the average year of publications. TIPS = transjugular intrahepatic portosystemic shunt.

Trebicka Jonel from Germany (46 articles), Hayes Peter C from the United Kingdom (38 articles), and Wang lei from China (37 articles) published the largest amount of papers in this field. Kamath Patrick S from USA (6676 citations), Bosch Jaime from Switzerland (4164 citations), and Ochs Andreas from Germany (2916 citations) were the top 3-cited authors (Table 3). The author collaboration network diagram indicates that several research teams had stable international scholarly communication, such as the team headed by Trebicka Jonel from University of Bonn (Germany), Han Guohong from The Fourth Military Medical University (China), and Bosch Jaime from University of Bern (Switzerland). In contrast, Xiong Bin from Huazhong University of Science and Technology (China) was the most active researcher in collaborative studies these years (Fig. 5).

**Table 3:** The top 10 authors involved in TIPS.

Rank	Author	Counts	Author	citations	Author	Total link strength
1	Trebicka Jonel (Germany)	46	Kamath Patrick S (USA)	6676	Han Guohong (China)	296
2	Hayes Peter C (UK)	38	Bosch Jaime (Switzerland)	4164	Trebicka Jonel (Germany)	271
3	Wang Lei (China)	37	Ochs Andreas (Germany)	2916	Fan Daiming (China)	251
4	Han Guohong (China)	36	Arroyo Vicente (Spain)	2563	Yin Zhanxin (China)	241
5	Redhead Doris N (UK)	35	Haag Kim (Germany)	2157	Niu Jing (China)	213
6	Gaba Ron C (USA)	35	Gerbes Alexander L (Germany)	2115	Meyer Carsten (Germany)	198
7	Bosch Jaime (Switzerland)	31	Rössle Martin (Germany)	2075	Praktiknjo Michael (Germany)	196
8	Meyer Carsten (Germany)	31	Laberge Jeanne M (USA)	1978	Guo Wengang (China)	196
9	Praktiknjo Michael (Germany)	30	Luca Angelo (Italy)	1930	Jansen Christian (Germany)	184
10	Fan Daiming (China)	28	Trebicka Jonel (Germany)	1861	Wang Lei (China)	184

TIPS = transjugular intrahepatic portosystemic shunt.

### 3.5. Journals publishing TIPS-related articles

As a cross-disciplinary area, roughly half TIPS-related articles were published in journals of gastroenterology and hepatology, and the other half were published in radiology-related journals. Six journals on the top 10 list of publication number were ranked in the top quartile of Journal Citation Reports 2024. Specifically, *Journal of Vascular and Interventional Radiology* (223 published, and 4814 cited) ranked first in publication number and fourth in citations. *Hepatology* (73 published, and 12,449 cited) published the highest-quality articles in this field (Table 4).

**Table 4:** The top 10 journals publishing the most articles on TIPS.

Rank	Journal	Documents	JCR	Journal	Citations	JCR
1	Journal of Vascular and Interventional Radiology	223	Q2	Hepatology	12,449	Q1
2	Cardiovascular and Interventional Radiology	151	Q2	Journal of Hepatology	7592	Q1
3	European Journal of Gastroenterology & Hepatology	116	Q3	Gastroenterology	6978	Q1
4	World Journal of Gastroenterology	81	Q1	Journal of Vascular and Interventional Radiology	4814	Q2
5	Hepatology	73	Q1	Radiology	4161	Q1
6	Radiology	70	Q1	American Journal of Gastroenterology	3293	Q1
7	American Journal of Gastroenterology	63	Q1	New England Journal of Medicine	2972	Q1
8	American Journal of Roentgenology	61	Q1	Gut	2705	Q1
9	Journal of Hepatology	59	Q1	American Journal of Roentgenology	2553	Q1
10	Digestive Diseases and Sciences	51	Q2	Cardiovascular and Interventional Radiology	2161	Q2

TIPS = transjugular intrahepatic portosystemic shunt.

### 3.6. Characteristics of top co-cited articles

The top 10 most co-cited articles were all published in top-ranked journals over 15 years ago. Of these, 4 were published in *The New England Journal of Medicine*, 5 were published in the journals of gastroenterology and hepatology, and one were published in *Radiology.* Except for 2 expert consensus statements,^[10,11]^ all these articles were clinical trials on the efficacy and safety of TIPS^[12–19]^ (Table 5). The 2 top co-cited articles were published in *The New England Journal of Medicine* by Rössle et al,^[12]^ and García-Pagán et al,^[13]^ respectively, both of which focused on TIPS treatment for variceal bleeding. Another 3 articles further investigated refractory ascites as a new indication for TIPS.^[14–16]^ Additionally, García-Pagán et al introduced early-TIPS as far back as 2010,^[13]^ which laid a foundation for the extension of TIPS indications. Furthermore, the research by Bureau et al provided high-quality evidence for the application of polytetrafluoroethylene-covered stent.^[17]^ Different from the early literature, the most co-cited articles in recent 5 years were published in specialty journals of gastroenterology and hepatology.^[20–22]^ Notably, all of these articles aimed to the extension of TIPS indications, which were considered as the current hotspot and future trend in this field.

**Table 5:** The top 10 references involved in TIPS.

Rank	Author	Year	Title	Journal	Citations
1	Rössle et al.	1994	The transjugular intrahepatic portosystemic stent-shunt procedure for variceal bleeding	N Engl J Med	400
2	García-Pagán et al.	2010	Early use of TIPS in patients with cirrhosis and variceal bleeding	N Engl J Med	390
3	LaBerge et al.	1993	Creation of transjugular intrahepatic portosystemic shunts with the wallstent endoprosthesis: results in 100 patients	Radiology	297
4	de Franchis et al.	2015	Expanding consensus in portal hypertension: Report of the Baveno VI Consensus Workshop: Stratifying risk and individualizing care for portal hypertension	J Hepatol	279
5	Malinchoc et al.	2000	A model to predict poor survival in patients undergoing transjugular intrahepatic portosystemic shunts	Hepatology	225
6	Boyer et al.	2010	The Role of Transjugular Intrahepatic Portosystemic Shunt (TIPS) in the Management of Portal Hypertension: update 2009	Hepatology	221
7	Bureau et al.	2004	Improved clinical outcome using polytetrafluoroethylene-coated stents for TIPS: results of a randomized study	Gastroenterology	213
8	Rössle et al.	2000	A comparison of paracentesis and transjugular intrahepatic portosystemic shunting in patients with ascites	N Engl J Med	213
9	Ochs et al.	1995	The transjugular intrahepatic portosystemic stent-shunt procedure for refractory ascites	N Engl J Med	212
10	Salerno et al.	2007	Transjugular intrahepatic portosystemic shunt for refractory ascites: a meta-analysis of individual patient data	Gastroenterology	211

TIPS = transjugular intrahepatic portosystemic shunt.

### 3.7. Keyword trends

A total of 3166 keywords were identified form the literature. The top 5 most frequently mentioned keywords were transjugular intrahepatic portosystemic shunt (681 times), portal hypertension (669 times), cirrhosis (435 times), TIPS (341 times), and liver cirrhosis (219 times), suggesting the definitive evidence of TIPS in the treatment of portal hypertension caused by cirrhosis. The next 10 frequently mentioned keywords mainly involved the indications and complications of TIPS, including ascites (197 times) and refractory ascites (69 times), variceal bleeding (147 times), Budd–Chiari syndrome (105 times), portal vein thrombosis (87 times), hepatorenal syndrome (54 times), and hepatic encephalopathy (164 times), etc (Table 6).

**Table 6:** The top 15 keywords involved in TIPS.

Rank	Keyword	Occurrences	Total link strength
1	Transjugular intrahepatic portosystemic shunt	681	1739
2	Portal hypertension	669	1898
3	Cirrhosis	435	1273
4	Tips	341	882
5	Liver cirrhosis	219	606
6	Ascites	197	680
7	Hepatic encephalopathy	164	486
8	Variceal bleeding	147	496
9	Liver transplantation	106	306
10	Budd-chiari syndrome	105	263
11	Portal vein thrombosis	87	267
12	Interventional radiology	84	226
13	Liver	81	268
14	Refractory ascites	69	222
15	Hepatorenal syndrome	54	207

TIPS = transjugular intrahepatic portosystemic shunt.

**Figure 6.**
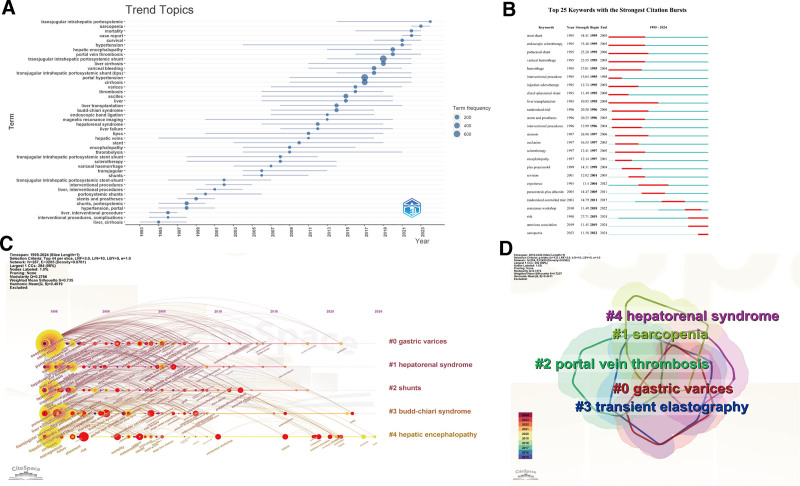
Analysis of trend topics and keywords in researches related to TIPS. (A) Trend topics analysis by Bibliometrix R-package. (B) Top 25 keywords with the strongest citation bursts by CiteSpace. (C) The temporal analysis of keyword clusters by CiteSpace. (D) The cluster analysis of keywords by CiteSpace. TIPS = transjugular intrahepatic portosystemic shunt.

Analysis of trend topics and keywords with the Strongest Citation Bursts might give us some clues of historic development, current hotspots and future direction in this field (Fig. 6A and B). The earliest studies mainly focused on efficacy comparisons between TIPS and endoscopic sclerotherapy for variceal bleeding. After about a decade of technical development, researchers turned attention to stent dysfunction and the combination of medication and intervention. Until the late 2010s, risk factors of prognosis became emerging hotspots. Particularly, as a prognostic factor, sarcopenia was probably the promising topic in the near future, since it was mentioned extremely frequently in recent 2 years.

The temporal analysis of keyword clusters reveals that hepatic encephalopathy was an ongoing concern over the past 3 decades (Fig. 6C). Spanning a period of the last decade (2015–2024), the identified keywords were divided into 5 clusters with close relationship by cluster analysis, which reflected the current hotspots and frontiers of this field (Fig. 6D). For instance, cluster #0 (varices), #2 (portal vein thrombosis), and #4 (hepatorenal syndrome) demonstrated the indication extending of TIPS. Cluster #3 (transient elastography) presented a novel diagnostic technique for cirrhotic portal hypertension. Remarkably, newly discovered prognostic factors, like sarcopenia (cluster #6), recently replaced the traditional scoring system, like odel for end-stage liver disease score (cluster #5), as the emerging focus of investigation (Fig. 6D).

## 4. Discussion

### 4.1. Progress and frontiers of TIPS

Annual publication trends typically reflect the changes of academic interest and attention in a research field. As mentioned above, the researches on TIPS underwent 3 stage of development which was linked to its clinical applications. Since Professor Richter first performed the procedure in Germany, TIPS had been rolled out worldwide within a short period, sparking a publication boom in this field.^[23]^ However, due to the high rates of stent dysfunction, publication in this field soon reached a plateau as the first round of clinical practice boom receded.^[24,25]^ Since 2013, studies on TIPS have been experiencing the renaissance fueled by the introduction of expanded polytetrafluoroethylene-covered stent, which significantly retarded shunt stenosis and occlusion, and improved clinical outcome of cirrhotic portal hypertension.^[17,26,27]^

Taken characteristics of top co-cited articles and keyword trends together, we found the scope of indications and management of complications were ongoing concerns in this field over the past decades. Spanning a period of the last decade, development of stent-graft, extension of indications, and prognosis of TIPS emerged as the current hotspot and future trend in this field. Notably, sarcopenia, as an independent keyword cluster, appeared in recent articles frequently.

### 4.2. Extension of indications

Integrating characteristics of top co-cited articles and temporal distribution of keyword clusters, we found researchers had been continuing searching for new indications of TIPS for decades. The indication of TIPS was initially limited to acute variceal bleeding, and soon extended to refractory ascites.^[14–16]^ A recent randomized controlled trial confirmed that TIPS with covered stents increase transplant-free survival of patients with cirrhosis and recurrent ascites.^[28]^ Driven by advances in stent-graft, complication management and patient stratification, both the timing and the indication of the procedure had been developing during the latest decade, such as introduction of early-TIPS and treatment for hepatic vascular diseases. The year 2010 became a turning point from “rescue TIPS” to “early TIPS” thanks to the landmark randomized controlled trial by Garcia-Pagan et al, which revealed that early TIPS significantly reduced treatment failure and mortality in patients with high risk of acute variceal bleeding.^[13]^ Subsequently, numerous similar clinical trials on extension of TIPS indication validated above conclusions and promoted the guideline updating together in recent years.^[2,29]^

### 4.3. Sarcopenia and prognosis

As illustrated in the map of trend topics and keywords with the Strongest Citation Bursts, sarcopenia emerged as the top hit among a mass of prognostic factors over the last decade. Growing literature provided suggestive evidence of association between sarcopenia and an increasing risk of post-TIPS hepatic encephalopathy and mortality.^[30–34]^ The leading hypothesis is that portal venous perfusion reduction after TIPS makes skeletal muscle become the primary organ for ammonia metabolism, and accordingly, sarcopenia contributes to elevated blood ammonia concentrations. Moreover, there were reports of prognostic models combined sarcopenia with classic scoring system, like model for end-stage liver disease score, Child-Pugh score, and Freiburg Index of Post-TIPS Survival, demonstrating improved predictive capability.^[35,36]^ More interestingly, TIPS may reverse sarcopenia to a certain degree, which would translate into a survival benefit in patients with portal hypertension.^[37,38]^ Therefore, the predictive value of sarcopenia for post-TIPS prognosis and the pathophysiological mechanisms behind sarcopenia reversal would probably become a novel research orientation in the near future.

## 5. Limitations

This study was limited by inclusion only of English-language publications from WoSCC, which may miss a small portion of literature. Then, low-frequency keywords were not taken into account, possibly overlooking some niche research frontiers.

## 6. Conclusion

This study provides profound insights into the evidences and perspectives of TIPS. Indication extension, optimal patient selection and the time of intervention have been the current hotspots in this field. Particularly, the association between TIPS and sarcopenia would become the main concerns in the near future.Supplemental digital content “Original download data” is available for this article (https://links.lww.com/MD/Q775).

## Author contributions

**Conceptualization:** Songwei Li.

**Data curation:** Kejia Li, Songwei Li.

**Formal analysis:** Kejia Li, Wenqing Zhang.

**Investigation:** Wenqing Zhang, Fan Yang, Lei Zhang.

**Methodology:** Kejia Li, Songwei Li.

**Project administration:** Songwei Li.

**Resources:** Songwei Li.

**Software:** Wenqing Zhang, Fan Yang.

**Supervision:** Songwei Li.

**Validation:** Kejia Li, Wenqing Zhang, Fan Yang, Lei Zhang.

**Writing – original draft:** Kejia Li, Songwei Li.

**Writing – review & editing:** Kejia Li, Wenqing Zhang, Fan Yang, Lei Zhang, Songwei Li.

## Supplementary Material


